# Broad-Spectrum Transgenic Resistance against Distinct Tospovirus Species at the Genus Level

**DOI:** 10.1371/journal.pone.0096073

**Published:** 2014-05-08

**Authors:** Jui-Chu Peng, Tsung-Chi Chen, Joseph A. J. Raja, Ching-Fu Yang, Wan-Chu Chien, Chen-Hsuan Lin, Fang-Lin Liu, Hui-Wen Wu, Shyi-Dong Yeh

**Affiliations:** 1 Department of Plant Pathology, National Chung Hsing University, Taichung, Taiwan; 2 Division of Crop Environment, Tainan District Agricultural Research and Extension Station, COA, Tainan, Taiwan; 3 Department of Biotechnology, Asia University, Wufeng, Taichung, Taiwan; 4 Agricultural Biotechnology Center, National Chung Hsing University, Taichung, Taiwan; 5 NCHU-UCD Plant and Food Biotechnology Center, National Chung Hsing University, Taichung, Taiwan; Department of Primary Industries and Fisheries, Australia

## Abstract

Thrips-borne tospoviruses cause severe damage to crops worldwide. In this investigation, tobacco lines transgenic for individual WLm constructs containing the conserved motifs of the L RNA-encoded RNA-dependent RNA polymerase (L) gene of *Watermelon silver mottle virus* (WSMoV) were generated by *Agrobacterium*-mediated transformation. The WLm constructs included: (i) translatable WLm in a sense orientation; (ii) untranslatable WLmt with two stop codons; (iii) untranslatable WLmts with stop codons and a frame-shift; (iv) untranslatable antisense WLmA; and (v) WLmhp with an untranslatable inverted repeat of WLm containing the tospoviral S RNA 3′-terminal consensus sequence (5′-ATTGCTCT-3′) and an *Nco*I site as a linker to generate a double-stranded hairpin transcript. A total of 46.7–70.0% transgenic tobacco lines derived from individual constructs showed resistance to the homologous WSMoV; 35.7–100% plants of these different WSMoV-resistant lines exhibited broad-spectrum resistance against four other serologically unrelated tospoviruses *Tomato spotted wilt virus*, *Groundnut yellow spot virus*, *Impatiens necrotic spot virus* and Groundnut chlorotic fan-spot virus. The selected transgenic tobacco lines also exhibited broad-spectrum resistance against five additional tospoviruses from WSMoV and Iris yellow spot virus clades, but not against RNA viruses from other genera. Northern analyses indicated that the broad-spectrum resistance is mediated by RNA silencing. To validate the L conserved region resistance in vegetable crops, the constructs were also used to generate transgenic tomato lines, which also showed effective resistance against WSMoV and other tospoviruses. Thus, our approach of using the conserved motifs of tospoviral L gene as a transgene generates broad-spectrum resistance against tospoviruses at the genus level.

## Introduction

Members of the genus *Tospovirus* are the only plant-infecting viruses in the family *Bunyaviridae*. Tospoviruses are transmitted by thrips in a persistent manner and they infect more than 1090 species in 85 families of monocots and dicots, causing severe damage in many economically important crops around the world [1], [2], [3]. The virion of tospovirus is an enveloped quasi-spherical particle with a tripartite RNA genome with five open reading frames [4]. The genomic segments are named after their size, as S (Small), M (Medium) and L (Large). The S RNA is ambisense and it encodes a nonstructural RNA-silencing suppressor protein (NSs) and the nucleocapsid protein (N) [5], [6]. The M RNA is also ambisense and it encodes a cell-to-cell movement protein (NSm) and the envelope glycoproteins precursor (GPp) [7], [8]. The L RNA is negative sense and it encodes an RNA-dependent RNA polymerase (RdRp), also called L protein [9].

The classification of different tospovirus species is based on the evolutionary genetic relationship of their N gene sequences, host ranges and vector specificity [10], [11]. Earlier, different tospovirus species were categorized into serogroups based on their serological similarities [10] determined by immunodetectable epitopes of N proteins and detecting antibodies. The tendency of the serogroups of tospoviruses to align with the clades defined by the phylogenetic analyses of tospoviruses has been observed in several of our studies [12], [13], [14], [15], [16]. Serological detection of tospoviruses is useful for easy on-the-spot diagnosis of tospoviruses [17].

The most effective and environmentally sound control strategies rely on the availability of resistant cultivars. However, due to high divergence of tospoviruses and scarcity of natural resistant resources, efficient control measures are difficult to be developed by traditional breeding. The *Sw-5* gene, first identified in tomato [18], [19], and *Tsw* gene in pepper [20] are widely used for resistance breeding against *Tomato spotted wilt virus* (TSWV). Unfortunately, several natural resistance-breaking strains of tospoviruses were reported all over the world, including in Australia, Brazil, Hawaii, Italy, South Africa, Spain, and USA [21], [22].

Since the concept of pathogen-derived resistance (PDR) was proposed [23] and confirmed by expressing the coat protein (CP) of *Tobacco mosaic virus* (TMV) in transgenic tobacco plants [24], transgenic resistance has become an important approach to protect various plant species against virus infection Transgenic tobacco plants that accumulated high levels of TSWV N protein exhibit broad-spectrum, but moderate level, resistance not only against the homologous isolate, but also against distantly related *Impatiens necrotic spot virus* (INSV) isolates [25]. However, the N protein-mediated protection can be overcome by increasing inoculum strength [26]. On the other hand, transgenic plants carrying untranslatable N [27], [28] or NSm gene [29] triggered RNA-mediated resistance to tospoviruses. Though RNA-mediated resistance provides higher degrees of resistance than protein-mediated resistance, it is specific against the homologous and closely related viruses [25], [27], [30], [31]. An artificial microRNA (amiRNA) approach targeting sequence elements within the conserved RdRp motifs of *Watermelon silver mottle virus* (WSMoV) L gene can successfully confer high degrees of transgenic resistance against the homologous virus [32]. To obtain resistance against multiple viruses, the N genes of TSWV, *Tomato chlorotic spot virus* (TCSV), and *Groundnut ringspot virus* (GRSV) were linked to generate transgenic tobacco plants with resistance to these three tospoviruses [33]. Similarly, a composite transgene containing small fragments from N genes of WSMoV, TSWV, GRSV and TCSV in a hairpin construct triggered RNA silencing for multiple resistances against the corresponding viruses [34]. Transgenic plants expressing an N protein-interacting peptide derived from the N open reading frame (ORF) of TSWV were also reported to confer high degrees of broad-spectrum resistance not only against TSWV, but also GRSV, and TCSV and Chrysanthemum stem necrosis virus (CSNV) [35]. However, N gene-mediated resistances, regardless of their direct origin from N transgenes [27], [28], [29], [33], [34] or from molecules (eg. peptides) targeting N gene sequences [35], may not be expected to be broad-spectrum and/or durable, because of the high degree of variation among the N gene sequences of tospoviral species and strains.

WSMoV, the type member of WSMoV clade [12], is one of the major limiting factors for cucurbit production in Taiwan [36] and other Asian countries [37], [38], [39]. The complete genome sequence of WSMoV has been determined [40], [41], [42], [43]. Recently, several new tospoviruses serologically related to WSMoV have been reported from India, China and East Asian countries [44], [45]. Comparison of the L protein sequence of WSMoV with those of other tospoviruses revealed a conserved region containing five RdRp motifs [43], [46], [47]. Based on the conserved region, genus-specific degenerate primers were designed for detecting most tospovirus species from greenhouse and field samples by reverse transcription-polymerase chain reaction (RT-PCR) [43], [48]. The objective of the present study was to develop broad-spectrum resistance against various tospoviral species using untranslatable transgenes designed from the highly conserved RdRp region of WSMoV L gene, through the post-transcriptional gene silencing (PTGS) mechanism.

Transformation of tobacco plants with the transgenes derived from the WSMoV L gene conserved region conferred broad-spectrum transgenic resistance not only against the WSMoV, but also against different tospovirus species from Asia type WSMoV, Iris yellow spot virus (IYSV) and *Groundnut yellow spot virus* (GYSV) clades and Euro-America type TSWV clade and INSV, which is considered as a distinct serotype [12]. When the same approach was extended to the real crop tomato, similar results were obtained. Thus, we conclude that the broad-spectrum resistance at the *Tospovirus* genus level generated by our approach is effective for the control of different tospovirus species infecting various crops.

## Materials and Methods

### Virus Sources

WSMoV [36] and Melon yellow spot virus (MYSV) [49] were collected from watermelon, and Peanut chlorotic fan-spot virus (renamed as Groundnut chlorotic fan-spot virus, GCFSV, by ICTV) [50] was collected from peanut in Taiwan. The high temperature-recovered isolate (HT-1) of Capsicum chlorosis virus (CaCV) collected from gloxinia in the United States was provided by H. T. Hsu [51]. TSWV NY, a New York isolate of TSWV, isolated from tomato was provided by R. Provvidenti, New York State Experiment Station, Geneva, Cornell University [42]. GRSV collected from tomato in Brazil [52] was provided by D. Gonsalves, Pacific Basin Agricultural Research Center, USDA, Hawaii. An isolate of INSV collected from impatiens in the United States [53] was provided by J. Moyer, North Carolina State University, USA. The iris isolate of IYSV from the Netherlands [54] and the tomato isolate of Tomato yellow ring virus (TYRV) from Iran [55] were provided by R. Kormelink, Wageningen University, The Netherlands (import permit: 96-V-54). Watermelon bud necrosis virus (WBNV), collected from watermelon in India [16], was provided by P. A. Rajagopalan, Mahyco Co., Jalna, India (import permit: 97-V-41). All these viruses were single lesion-isolated in the local lesion host *Chenopodium quinoa* Willd. and maintained in the systemic host *Nicotiana benthamiana* Domin. by mechanical inoculation under temperature-controlled (23–28°C) greenhouse conditions. Virus inocula were prepared by grinding infected leaves in 10 mM potassium phosphate buffer (pH 7.0) containing 10 mM sodium sulfite (1∶10 w/v).

Other viruses not belonging to the genus *Tospovirus,* used for comparison, include *Zucchini yellow mosaic virus* (ZYMV) TN-3 isolate [56], maintained in squash plants, *Papaya ringspot virus* (PRSV) YK isolate [57], maintained in papaya (*Carica papaya* L. cv. Tainung No. 2) plants, and *Turnip mosaic virus* (TuMV) CY-5 isolate [58] and *Cucumber mosaic virus* (CMV) [48] both maintained in *N. benthamiana* plants. One week after mechanical inoculation, inocula of these viruses were prepared by grinding infected leaf tissues in 10 mM potassium phosphate buffer (pH 7.2, 1∶20 w/v).

### Sequence Alignment

Sequences of the reported full-length L RNAs for the comparison were obtained from GenBank database, through the following accession numbers: TSWV, (NC_002052); INSV, (NC_003625); *Groundnut bud necrosis virus* (GBNV), (NC_003614); WSMoV, (NC_003832); CaCV, (NC_008302); and MYSV, (NC_008306). Nucleotide sequences were compared by BestFit program of Seqweb v. 3.1 (Accelrys, Inc., San Diego, CA). Multiple sequence alignment of L genes was carried out using ClustalW+ program of Seqweb v. 3.1.

### Construction of Translatable and Untranslatable Constructs Containing WSMoV L Gene Conserved Region

Total RNAs were isolated from leaves of the WSMoV-infected *N. benthamiana* plants by Ultraspec RNA isolation system (Biotex laboratories, Houston, TX). The primers WL3975(*Nco*I) (5′-GCCATGGAGCACACATACAAGCATATCGCC-3′, *Nco*I site underlined) and WL4928c(*Sac*I) (5′-GAGCTCGAGTCGTTCTCTTCTCCTGGCAGC-3′, *Sac*I site underlined) were used to amplify the nt 3975–4928 region of the vc strand of WSMoV L RNA, corresponding to the L gene conserved region containing five RdRp motifs, by RT-PCR. The amplified fragment, denoted WLm, was cloned in TOPO TA vector (Invitrogen, Carlsbad, CA), and after verifying the sequence, WLm was used as the template for subsequent modifications.

**Figure 1:**
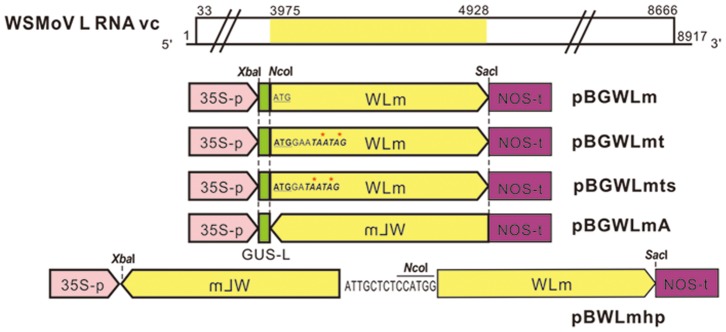
Schematic representation of individual transgenes. Nucleotide (nt) 3975 to 4928 region of the viral complementary (vc) strand of L RNA (yellow box) of *Watermelon silver mottle virus* (WSMoV) was amplified as the WLm fragment and constructed as translatable sense (pBGWLm), non-translatable sense (pBGWLmt and pBGWLmts), antisense (pBGWLmA) and inverted repeat (pBWLmhp) transgenes in the Ti binary vector pBI121. pBGWLmt and pBGWLmts contained identical sequence to pBGWLm, but two termination codons (italicized and asterisked) were added in-frame in pBGWLmt, and the same were added with a −1 frame shift in pBGWLmts. A linker sequence containing the tospoviral S RNA 3′-terminal consensus sequence (5′-ATTGCTCT-3′) and an *Nco*I site was added between antisense and sense WLm sequences for creating the inverted repeat in pBWLmhp. The restriction sites used for construction of transgenes are indicated. GUS leader sequence (GUS-L, green box), *Cauliflower mosaic virus* 35S promoter (35S-p, pink box) and *nos* terminator (NOS-t, purple box) are indicated.

The primers WL3975*Nco*I (5′-GCCATGGAA*TAATAG*GAGCACACATACAAGCATATCGCC-3′, *Nco*I site underlined and two termination codons italicized) and WLst3975*Nco*I (5′-GCCATGGA*TAATAG*GAGCACACATACAAGCATATCGCC-3′, *Nco*I site underlined, frame-shifted termination codons italicized) individually coupled with WL4928c*Sac*I were used to introduce two in-frame termination codons and two termination codons with a −1 frameshift at the 5′ end of the WLm fragment to generate the untranslatable fragments of WLmt and WLmts, respectively. Primers WL4928c*Nco*I (5′-CCATGGGTCGTTCTCTTCTCCTGGCAGC-3′, *Nco*I site underlined) and WL3975*Sac*I (5′-GAGCTCGAGCACACATACAAGCATATCGCC-3′, *Sac*I site underlined) were used to amplify WLm, containing opposite cloning sites for generating antisense fragment WLmA. The WLm, WLmt, WLmts and WLmA fragments were subsequently cloned into the plasmid pBGCP [59], a derivative of the binary vector pBI121 carrying the coat protein (CP) gene of PRSV with a *β*-glucuronidase (GUS) leader sequence, via *Nco*I and *Sac*I sites, to generate pBGWLm, pBGWLmt, pBGWLmts and pBGWLmA, respectively (Figure 1).

Furthermore, a WLm-linker fragment amplified by the primers WL4928c*Xba*I (5′-ATGCTCTAGAGTCGTTCTCTTCTCCTGGCAGC-3′, *Xba*I site underlined) and WL3975*Nco*-linker [5′-CCGGCCATGG *AGAGCAAT*GAGCACACATACAAGCA-3′ with an *Nco*I site (underlined) and a stretch of eight nucleotides (italicized) complementary to the tospoviral S RNA 3′-terminal consensus sequence] was cloned in TOPO TA vector. The S RNA 3′-terminal consensus sequence was included in the linker for further increasing the effectiveness of the transgene. The *Xba*I/*Nco*I-digested WLm-linker and *Nco*I/*Sac*I-digested WLm fragments were mixed with *Xba*I/*Sac*I-digested pBI121 for ligation to generate the construct pBWLmhp (Figure 1) that expressed a hairpin RNA from the inverted repeat transgene WLmhp.

Finally, all the transgene constructs were transferred into the *Agrobacterium tumefaciens* strain LBA 4404 by direct transformation with liquid nitrogen treatment [60].

### Tobacco and Tomato Transformation

*A. tumefaciens* LBA 4404 containing individual constructs were used to transform tobacco plants of *N. benthamiana*, using small leaf pieces as described by Horsch *et al*., [61]. The plants of each line were originated and cloned from a single independent regenerated shoot.

For tomato transformation, the cotyledon explants were prepared from 14-day-old seedlings germinated from manually peeled and surface sterilized seeds of *Solanum lycopersicum* L. cv. Feminini Beauty (Evergrow Seed Co. Ltd., Taiwan) on half strength MS medium [62]. The explants were further pre-cultured on MS medium for two days. The pre-cultured cotyledon discs were infected with 1∶15 dilution of overnight grown *Agrobacterium* culture to introduce the individual transgene constructs by *Agrobacterium*-mediated transformation, as previously described [63]. Multiple shootlets micropropagated from a single shoot regenerated on the selection medium were cut and transferred to the rooting medium (MS medium with 0.5 mg/l IBA and 50 mg/l kanamycin) and defined as shootlets of a R_0_ line. After 1 wk, the rooted plantlets were transferred to vermiculite in plastic bags and maintained in growth chamber at 25–27°C with 16 hr photoperiod (53 µE m^−2^ s^−1^ photon irradiance). After acclimatization for 2 wk, the plantlets were transferred to a temperature-controlled (23–28°C) greenhouse.

### Examination of Putative Transgenic Lines

To check the presence of individual transgenes in the generated transgenic *N. benthamiana* and tomato plants, total genomic DNA was extracted from leaves of non-transformed or transgenic plants by Genomic DNA Purification Kit (GMbiolab, Taichung, Taiwan), according to manufacturer’s instructions. The primer pair WL3975(*Nco*I)/WL4928c(*Sac*I) was used for the transgenic *N. benthamiana* and tomato lines derived from pBGWLm, pBGWLmt, pBGWLmts, pBGWLmA and pBWLmhp. Primers PNPTII (5′-ATGATTGAACAAGATGGATTGCAC-3′) and MNPTII (5′-GAAGAACTCGTCAAGAAGGCGATA-3′) were designed to check the presence of the selection-marker neomycin phosphotransferase (*npt*II) gene linked with the transgenes in all transgenic plants. Fifty nanogram of extracted DNAs were used as templates, and PCR was conducted with 1 min for denaturation at 94°C, 2 min for annealing at 58°C (except 50°C for transgenic plants derived from pBWLmhp), and 3 min for synthesis at 72°C for 34 cycles, followed by a final extension at 72°C for 7 min. PCR products were analyzed by electrophoresis on 1% agarose gel.

### Evaluation of Resistance to Homologous and Heterologous Tospoviruses

To evaluate the transgenic resistance to the homologous tospovirus, non-transgenic and putative transgenic *N. benthamiana* and tomato plants were mechanically inoculated with WSMoV under temperature controlled (23–28°C) greenhouse conditions. To evaluate the transgenic resistance against a heterologous tospovirus, the selected WSMoV-resistant transgenic tobacco R_0_ lines multiplied *in vitro* were mechanically inoculated with TSWV. To further evaluate the spectrum of the transgenic resistance, the *in vitro* multiplied WSMoV-resistant transgenic tobacco and tomato R_0_ lines were mechanically inoculated with individual tospoviruses which represent different clades of tospoviruses, including TSWV, GRSV, GCFSV and INSV, under the same greenhouse conditions. All inocula for challenging transgenic plants were prepared from TSWV, WSMoV, GRSV, INSV or GCFSV-infected *N. benthamiana* leaves, ground and diluted 50 to 100-fold (w/v) in 10 mM potassium phosphate buffer (pH 7.0) containing 10 mM sodium sulfite. Five plants of each transgenic line were inoculated with each tospovirus. Individual inocula were applied by rubbing the two fully-expended leaves with 600 mesh carborundum at the stage of 4–5 leaves. Because tospoviruses are not stable, for each inoculum, non-transgenic control plants were inoculated after inoculating the transgenic lines to ensure the infection. In some cases, the plants were inoculated twice with an interval of one week between the two inoculations. The plants were observed for 30 days for symptom development under the aforementioned greenhouse conditions.

For test the spectrum of resistance, the selected WSMoV-resistant transgenic tobacco lines were further mechanically challenged with other Asia-type tospoviruses, including WBNV, MYSV, TYRV, IYSV and CaCV, following the method described above. TuMV and CMV were used as out-group viruses for challenging the transgenic tobacco lines which exhibited broad-spectrum resistance against tospoviruses.

### Indirect Enzyme-linked Immunosorbent Assay (ELISA)

Indirect ELISA was conducted following the method described by Yeh and Gonsalves [64] with required modifications. Leaf extracts prepared from four leaf disks (0.5 cm diameter) punched from different upper leaves of the tested plants 30 days post inoculation (dpi) were ground in coating buffer (50 mM sodium carbonate, pH 9.6, containing 0.01% sodium azide) at 1∶40 dilution and used for antigen coating. The antisera against the N protein of WSMoV, TSWV, GRSV, INSV or GCFSV [50] were used at a 1∶4000 dilution in conjugate buffer (PBS containing 0.05% Tween 20, 2% polyvinylpyrrolidone-40 and 0.2% ovalbumin). The alkaline phosphatase-conjugated goat anti-rabbit IgG (Jackson ImmunoResearch Laboratories, West Grove, PA) was used at 1∶5000 dilution as the secondary antibody. The incubation times for the reaction of crude antigens with the primary antibody and the reaction of rabbit IgG with the secondary antibody were both set at 37°C for 60 min. The absorbance at 405 nm was recorded using the Victor 1420 Multilabel Counter (PerkinElmer Life Sciences, Waltham, MA) 30 min after the addition of *ρ*-nitrophenyl phosphate substrate (Sigma-Aldrich, St. Louis, MO) that was dissolved in substrate buffer (9.7% diethanolamine and 0.02% sodium azide, pH 9.8). The threshold of a positive reaction was set at two-fold readings, as compared to that of the negative control.

### Transcript and siRNA Detection by Northern Hybridization

For transgene transcript detection, total RNAs were isolated from leaves of non-transgenic or transgenic plants by Ultraspec RNA isolation system (Biotex laboratories) according to manufacturer’s instructions. Twenty microgram of total RNAs were separated in 1.0% agarose gels with formaldehyde, and then transblotted onto Hybond-N+ nylon membrane (Amersham Biosciences, Buckinghamshire, UK). After UV-crosslinking of the transferred RNAs to the nylon membrane, hybridization was done with the ^32^P-labeled DNA probe prepared from WLm by Primer-It II Random Primer Labeling Kit (Stratagene, La Jolla, CA). For siRNA detection, northern hybridization of total RNAs were performed as described previously [65], [66]. The hybridization signals were detected by autoradiography.

### Southern Hybridization

Total genomic DNAs were extracted from leaves of non-transgenic or transgenic *N. benthamiana* plants by Genomic DNA Purification Kit (GMbiolab, Taichung, Taiwan), according to manufacturer’s instructions. Fifteen to twenty microgram of genomic DNAs were digested with *Ssp*I, separated at 120 V in a 0.8% agarose gel, transblotted onto Hybond-N+ nylon membrane (Amersham Pharmacia Biotech, UK), and hybridized with the ^32^P-labeled probe, as described above. The hybridization signals were detected by autoradiography.

## Results

### Relationship of WLm within L RNAs of Distantly Related Tospoviruses

**Figure 2:**
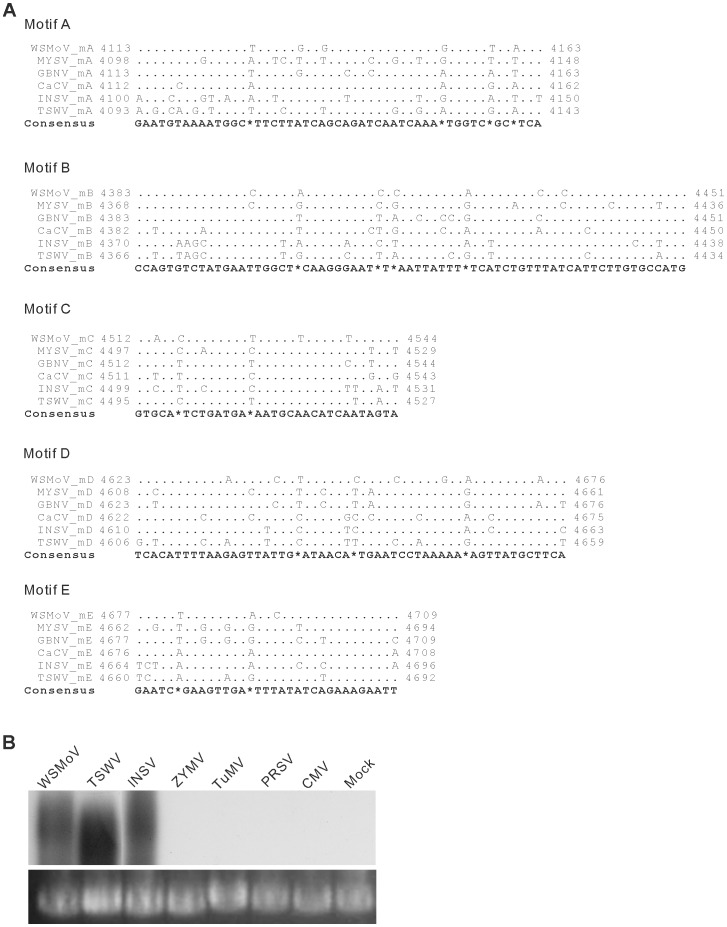
Comparison of tospoviral L gene region encoding RNA-dependent RNA polymerase (RdRp) conserved motifs of L protein. (A) Alignment of L gene sequences corresponding to the RdRp motifs, A, B, C, D and E of L proteins of *Watermelon silver mottle virus* (WSMoV), Capsicum chlorosis virus (CaCV), *Groundnut bud necrosis virus* (GBNV), Melon yellow spot virus (MYSV), *Tomato spotted wilt virus* (TSWV) and *Impatiens necrotic spot virus* (INSV). (B) The recognizability of the L RNAs of WSMoV, TSWV and INSV by the ^32^P-labeled probe prepared using the cloned L gene conserved region (WLm), as analyzed by northern hybridization. Genomic RNAs of *Zucchini yellow mosaic virus* (ZYMV), *Turnip mosaic virus* (TuMV), *Papaya ringspot virus* (PRSV) and *Cucumber mosaic virus* (CMV) were also used in the hybridization analysis. RNA from mock inoculated plant was used as the negative control. Ribosomal RNAs indicate the loading controls.

The sequence of the conserved region containing five RdRp motifs, motif A to E, of WSMoV was compared with those of TSWV, INSV, CaCV, GBNV and MYSV. This conserved region, amino acid (aa) 1315 to 1632 of WSMoV L protein, shares identities of 93.7%, 95.9%, 96.9%, 72.6% and 66.7% with those of MYSV, CaCV, GBNV, INSV and TSWV, respectively. The corresponding nucleotide sequence, nt 3975 to 4928 of the vc strand of WSMoV L RNA, shares identities of 82.9%, 82.6%, 79.4%, 69.0% and 66.4% with those of CaCV, GBNV, MYSV, TSWV and INSV, respectively (Table 1). High aa and nt identities of 76.5–100% and 75.5–93.8%, respectively, were found among the RdRp motifs of the tospoviruses compared (Table 1). Alignments of the nt sequences encoding individual RdRp motifs of WSMoV, GBNV, CaCV, MYSV, TSWV and INSV are shown in Figure 2A.

**Table 1:** Nucleotide (nt) and amino acid (aa) identities of the L conserved region, viral complementary position of the nt 3975 to 4928 of L RNA, containing five RNA-dependent RNA polymerase motifs of *Watermelon silver mottle virus* (WSMoV) compared with those of other tospoviruses.

Virus	Conserved region		RdRp motif									
			A		B		C		D		E	
	nt (%)	aa (%)	nt (%)	aa (%)	nt (%)	aa (%)	nt (%)	aa (%)	nt (%)	aa (%)	nt (%)	aa (%)
CaCV	82.9	95.9	88.2	100	84.1	100	81.3	90.9	77.8	100	93.8	100
GBNV	82.6	96.9	88.2	100	87.0	100	84.9	100	85.0	100	81.3	100
MYSV	79.4	93.7	80.4	100	89.9	100	81.3	100	79.6	100	81.8	100
TSWV	69.0	66.7	80.0	76.5	78.3	100	84.9	81.8	78.8	88.9	83.9	90.9
INSV	66.4	72.6	75.5	82.4	81.2	100	78.1	81.8	83.0	94.4	86.2	90.9

Sequences of tospoviral L RNAs for the comparison were obtained from GenBank: WSMoV, NC_003832; *Tomato spotted wilt virus* (TSWV), NC_002052; *Impatiens necrotic spot virus* (INSV), NC_003625; *Groundnut bud necrosis virus* (GBNV), NC_003614; Capsicum chlorosis virus (CaCV), NC_008302; and Melon yellow spot virus (MYSV), NC_008306.

To confirm the relationship among the conserved region of L genes of tospoviruses, the construct WLm was used as a template for preparation of ^32^P-labeled DNA probes to hybridize with total RNAs extracted from leaf tissues of plants infected with various plant viruses, including WSMoV, TSWV and INSV belonging to the genus *Tospovirus*; TuMV, ZYMV and PRSV belonging to the genus *Potyvirus*; and CMV belonging to the genus *Cucumovirus*. Strong hybridization signals were observed from RNA prepared from WSMoV-infected plants. Comparably strong signals were also observed from RNA prepared from plants infected by TSWV or INSV that are serologically unrelated to WSMoV, suggesting that WLm-derived probes derived from the construct WLm are able to react with the conserved regions of L genes of TSWV and INSV. Corroboratively, no hybridization signals were detected from the RNAs prepared from TuMV, ZYMV, PRSV, or CMV-infected plant and the negative control of mock-inoculated plant (Figure 2B). Based on the results of sequence analysis (Figure 2A) and hybridization analysis (Figure 2B), the region spanning nt 3975 to 4928 of the vc strand of WSMoV L RNA (denoted WLm) was used for construction of various transgenes targeting the conserved RdRp motifs of tospovirus L genes to trigger transgenic resistance.

### Resistance to Homologous WSMoV in Transgenic Tobacco Lines Triggered by Various Transgene Constructs

*N. benthamiana* plants were transformed using the five constructs, pBGWLm, pBGWLmt, pBGWLmts, pBGWLmA and pBWLmhp (Figure 1), carrying transgenes derived from the conserved region of WSMoV L gene. The GUS leader sequence of PB1121 was retained also at the 5′-terminal region of the untranslatable transgenes in the constructs pBGWLmt, pBGWLmts and pBGWLmA to maintain the translation capability similar to that of the translatable pBGWLm. Different categories of transgenic *N. benthamiana* lines carrying different transgenes were denoted by the names of the transgenes they carried (i.e., WLm, WLmt, WLmts, WLmA or WLmhp) and individual lines/plants from each category were identified by numeral(s) following their category name.

After kanamycin selection and PCR detection, the putative transgenic *N. benthamiana* R_0_ lines, 30 lines for each construct, were first evaluated for resistance to WSMoV and the results are summarized in Table 2. The transgenic lines which exhibited delay in symptom development for more than 7 days relative to the non-transgenic control, and also ELISA positive for WSMoV N protein were classified as moderately resistant (MR) lines; while those that did not show any symptoms one month after inoculation, and were ELISA negative were classified as highly resistant (HR) lines. Fourteen WLm lines (46.7%), 19 WLmt lines (63.3%), 19 WLmts lines (63.3%), 20 WLmA lines (66.7%) and 21 WLmhp lines (70.0%) exhibited resistance to WSMoV. Among them, 30.0–40.0% (9–12 lines) of resistant lines were HR type to WSMoV infection and 13.3–40.0% (4–12 lines) were MR type (Table 2). The results indicated that each of the five transgene constructs was able to trigger high levels of resistance to WSMoV in *N. benthamiana* transgenic lines. However, the non-translatable and inverted repeat transgene constructs WLmt, WLmts, WLmA and WLmhp rendered higher protection efficiencies (63.3% to 70.0%) compared to the translatable transgene construct WLm (46.7%) (Table 2), indicating that RNA-mediated resistance plays a key role.

**Table 2:** Evaluation of transgenic *Nicotiana benthamiana* R_0_ lines carrying individual constructs containing L gene conserved region for resistance to the homologous *Watermelon silver mottle virus* (WSMoV) under greenhouse conditions.

Transgene	Total assayed line No.	Challenged withWSMoV			
		HR^a^	MR^b^	S^c^	Total R lines (%)^d^
WLm	30	10	4	16	14 (46.7)^A^ *
WLmt	30	12	7	11	19 (63.3)^B^
WLmts	30	12	7	11	19 (63.3)^B^
WLmA	30	11	9	10	20 (66.7)^B^
WLmhp	30	9	12	9	21 (70.0)^B^

a Transgenic *N. benthamiana* lines that remained asymptomatic and ELISA negative for WSMoV NP beyond a month after inoculation were considered highly resistant (HR) lines.

b Transgenic *N. benthamiana* lines that showed more than 7 days delay in symptom development in comparison to the non-transformed plants were classified as moderately resistant (MR) lines.

c Transgenic *N. benthamiana* lines that exhibited typical systemic symptoms similar to non-transformed *N. benthamiana* plants, at the same time or less than 7 days delay after inoculation with WSMoV, were regarded as susceptible (S) lines.

d Both HR and MR lines were considered as resistant (R) lines. The percentages of resistance were expressed as number of R lines/total number of total assayed lines.

*A & B: adjacent values that are superscribed by same alphabet are not significantly different as analyzed by chi-square using software JMP 5.0.1 (SAS Institute Inc.) (*p*<0.05).

### The L Gene Conserved Region of WSMoV Confers Broad-spectrum Resistance Against Serologically Unrelated Tospoviruses in Transgenic Tobacco Plants

Subsequently, the WSMoV-resistant transgenic *N. benthamiana* lines were challenged with serologically unrelated tospovirus species of TSWV, GRSV, INSV and GCFSV, to further evaluate the spectrum of the resistance. The results are presented in Table 3. The virus infection of symptomatic transgenic plants was confirmed by indirect ELISA using the antiserum against the N protein of TSWV, GRSV, INSV or GCFSV. If no significant accumulation of the virus was detected by ELISA one month after inoculation, the resistant transgenic plants were considered immune to virus infection. Of the 14 WSMoV-resistant WLm transgenic lines, 35.7–71.4% exhibited different levels of broad-spectrum resistance. Most WSMoV-resistant lines showed different levels of resistance against 2 to 5 different challenged tospovirus species, except WLm-16 that was resistant only to WSMoV. Especially, the lines WLm-11 and WLm-30 were resistant to all the tested tospovirus species. WLm-11 was immune to INSV, but developed delayed symptoms when challenged with TSWV, GRSV and GCFSV. WLm-30 was immune to GRSV and GCFSV, but developed delayed symptoms when challenged with TSWV and INSV.

**Table 3:** Evaluation of the *Watermelon silver mottle virus* (WSMoV)-resistant transgenic *Nicotiana benthamiana* lines against other serologically unrelated tospovirus species, *Tomato spotted wilt virus* (TSWV), *Groundnut ringspot virus* (GRSV), *Impatiens necrotic spot virus* (INSV) and Groundnut chlorotic fan-spot virus (GCFSV) under greenhouse conditions.

Transgene	Total assayed line No.	TSWV			GRSV			INSV			GCFSV		
		HR^a^	MR^b^	Total R lines (%)^c^	HR	MR	Total R lines (%)	HR	MR	Total R lines (%)	HR	MR	Total R lines (%)
WLm	14	0	6	6 (42.9)	2	5	7 (50.0)	2	3	5 (35.7)	4	6	10 (71.4)
WLmt	19	4	6	10 (52.6)	4	6	10 (52.6)	5	6	11 (57.9)	7	7	14 (73.7)
WLmts	19	7	4	11 (57.9)	4	9	13 (68.4)	7	6	13 (68.4)	9	10	19 (100.0)
WLmA	20	5	5	10 (50.0)	6	5	11 (55.0)	2	9	11 (55.0)	5	10	15 (75.0)
WLmhp	21	6	9	15 (71.4)	2	13	15 (71.4)	9	7	16 (76.2)	6	10	16 (76.2)

a The lines that remained asymptomatic and ELISA negative for the N proteins beyond a month after inoculation were considered highly resistant (HR) lines.

b The lines that showed more than 7 days delay in symptom development, in comparison to the non-transformed plants were classified as moderately resistant (MR) lines.

c Both HR and MR lines were regarded as resistant (R) lines. The percentages of resistance were expressed as number of R lines/total number of assayed WSMoV-resistant lines.

**Figure 3:**
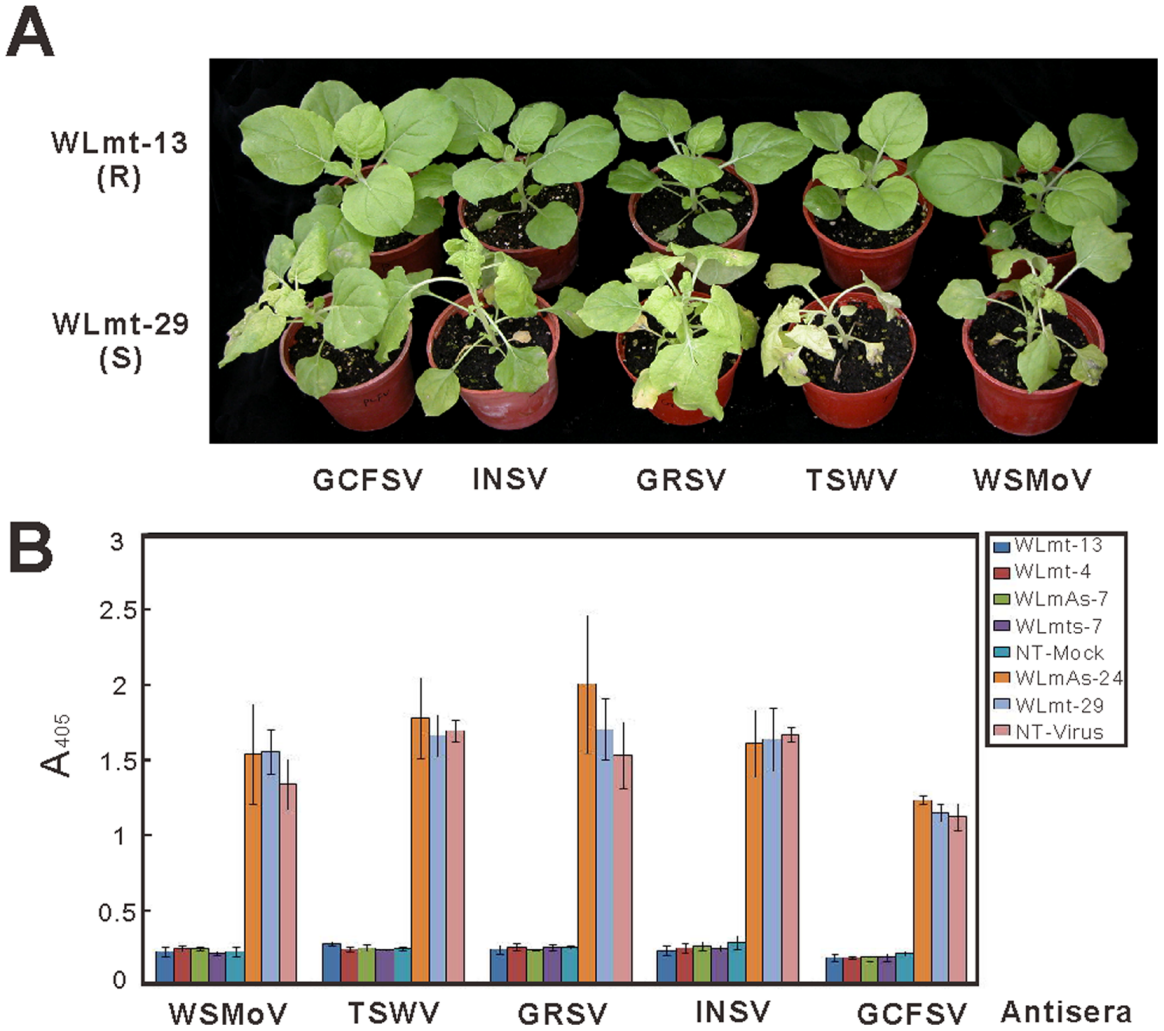
Evaluation of the transgenic *Nicotiana benthamiana* lines for resistance to different tospovirus species under greenhouse conditions. The lines with individual constructs of the conserved region of L gene of *Watermelon silver mottle virus* (WSMoV) were tested. (A) Resistant (R) line WLmt-13 exhibited broad-spectrum resistance to five tospovirus species, WSMoV, *Tomato spotted wilt virus* (TSWV), *Groundnut ringspot virus* (GRSV), *Impatiens necrotic spot virus* (INSV) and Groundnut chlorotic fan-spot virus (GCFSV). Susceptible (S) line WLmt-29 showed typical systemic symptoms similar to the non-transformed control after inoculation with individual tospoviruses was used for comparison. (B) Virus infections were confirmed by indirect enzyme-linked immunosorbent assay (ELISA) using the antiserum against the nucleocapsid protein of WSMoV, TSWV, GRSV, INSV or GCFSV. ELISA readings were recorded from the average of three independent experiments. Fourteen days post inoculation, the symptoms were photographed and ELISA was conducted.

Among 19 WSMoV-resistant WLmt transgenic lines, 52.6–73.7% of them showed different levels of resistance to TSWV, GRSV, INSV and GCFSV. Six lines, WLmt-4, 5, 7, 9, 13 and 17, were resistant, at various levels, to all the five challenged tospoviruses. Indeed, two of them, WLmt-4 and WLmt-13, were immune to all challenged tospoviruses; from the asymptomatic plants of these lines, virus accumulation was not detectable by indirect ELISA (Figure 3).

Among the 19 WSMoV-resistant WLmts transgenic lines, 57.9–100% of them exhibited different levels of resistance to TSWV, GRSV, INSV and GCFSV. Seven lines, WLmts-2, 5, 7, 10, 13, 16 and 24, were resistant to all challenged tospoviruses with different levels of resistance. Particularly, WLmts-7 was immune to all challenged tospoviruses, similar to WLmt-13 (Figure 3). Among the 20 WSMoV-resistant WLmA transgenic lines, 50–75% of them exhibited different degrees of resistance to TSWV, GRSV, INSV and GCFSV. Five lines, WLmA-7, 8, 19, 20 and 21, showed different levels of resistance to the challenged tospoviruses. Particularly, WLmA-7 was immune to these challenged viruses, similar to WLmt-13 (Figure 3).

Moreover, in 21 WSMoV-resistant WLmhp transgenic lines, 71.4–76.2% of the plants showed different levels of broad-spectrum resistance to TSWV, GRSV, INSV and GCFSV. Seven lines, WLmhp-2, 4, 10, 15, 16, 22 and 24, exhibited different levels of resistance to all the challenged tospoviruses. However, no line was immune to any of those viruses.

**Figure 4:**
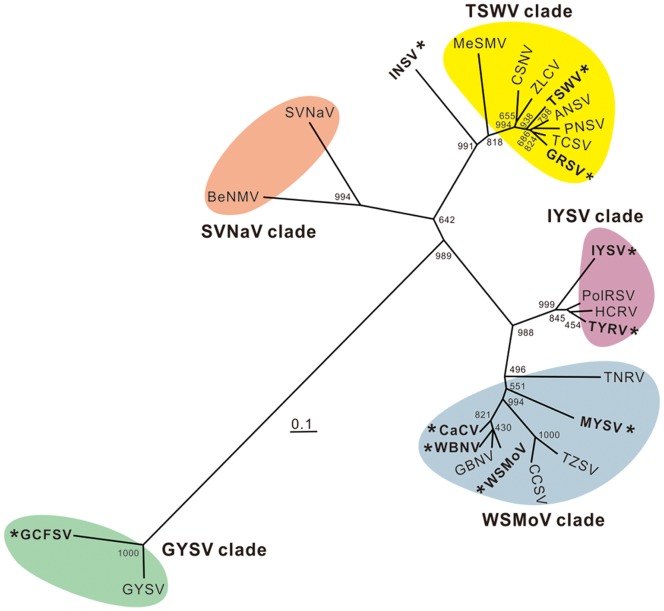
Phylogenetic relationship of tospoviruses based on the sequences of nucleocapsid (N) proteins. The dendrogram was generated using the Neghbour-Joining algorithm based on 1000 bootstrap replicates. Viruses sharing close serological relationship in their N proteins are highlighted and designated as a clade. The viruses used to evaluate the transgenic resistance in this investigation are asterisked. The sequences of N genes were retrieved from GenBank of National Center for Biotechnology Information. ANSV, Alstroemeria necrotic streak virus (GQ478668); BeNMV, Bean necrosis mosaic virus (NC_018071); CaCV, Capsicum chlorosis virus (NC_008301); CCSV, Calla lily chlorotic spot virus (AY867502); CSNV, Chrysanthemum stem necrosis virus (AB600873); GBNV, *Groundnut bud necrosis virus* (U27809); GCFSV, Groundnut chlorotic fan-spot virus (AF080526); GRSV, *Groundnut ringspot virus* (L12048); GYSV, *Groundnut yellow spot virus* (AF013994); HCRV, Hippeastrum chlorotic ringspot virus (KC290943); INSV, *Impatiens necrotic spot virus* (NC_003624); IYSV, Iris yellow spot virus (AF001387); MeSMV, Melon severe mosaic virus (EU275149); MYSV, Melon yellow spot virus (AB038343); PNSV, Pepper necrotic spot virus (HE584762); PolRSV, Polygonum ringspot virus (EF445397); SVNaV, Soybean vein necrosis-associated virus (JF808214); TCSV, *Tomato chlorotic spot virus* (S54325); TNRV, Tomato necrotic ringspot virus (FJ489600); TSWV, *Tomato spotted wilt virus* (D13926); TYRV, Tomato yellow ring virus (AY686718); TZSV, Tomato zonate spot virus (NC_010489); WBNV, Watermelon bud necrosis virus (EU249351); WSMoV, *Watermelon silver mottle virus* (U78734); and ZLCV, *Zucchini lethal chlorosis virus* (AF067069).

Taken together, our results demonstrated that the L gene conserved region of WSMoV is able to trigger resistance not only against the homologous WSMoV, but also against other serologically unrelated tospoviruses, such as TSWV, GRSV, INSV and GCFSV, in transgenic *N. benthamiana* plants. Four particular lines, WLmt-4, WLmt-13, WLmts-7 and WLmA-7, showed complete resistance to the four heterologous tospovirus species. In further tests, these four tobacco lines also provided complete resistance against WBNV, MYSV, TYRV, IYSV and CaCV (data not shown). The 10 tospoviruses species used for challenge inoculation belong to different clades of tospoviruses: CaCV, WBNV, WSMoV and MYSV from WSMoV clade; TSWV and GRSV from TSWV clade; IYSV and TYRV from IYSV clade; GCFSV from GYSV clade; and INSV (Figure 4).

When RNA viruses not belonging to the genus *Tospovirus* such as TuMV (*Potyvirus*) and CMV (*Cucumovirus*) were used to challenge the four lines immune to tospoviruses, the plants showed full susceptibility similar to the non-transgenic controls. Thus, our results suggest that the broad-spectrum resistance occurs at the genus level specifically to *Tospovirus*.

### The L Gene Conserved Region Provides Broad-spectrum Resistance to WSMoV, TSWV and GRSV in Transgenic Tomato Plants

Nearly 80–90 independent lines of transgenic tomato plants were regenerated after transformation with each construct of pBGWLm, pBGWLmt, pBGWLmts, pBGWLmA and pBWLmhp. Following the selection on the kanamycin medium, plants of individual R_0_ lines were transferred to the greenhouse and challenged with a virulent TSWV isolate. A remarkably high number of 38–40% resistance of all WLmhp and WLmA lines showed high or moderate resistance to TSWV infection (Table 4). From almost all cases in our previous results of transgenic tobacco, we noticed that when a tomato line was resistant to the heterologous unrelated TSWV, it was also resistant to the homologous WSMoV and GRSV. The results for the WLm, WLmt and WLmts lines were less consistent. The resistance conferred by these three constructs was lower for TSWV and other tospoviruses (Table 4).

**Table 4:** Evaluation of transgenic tomato (*Solanum lycopersicum* L. cv. Feminini Beauty) lines, which carry individual constructs containing the conserved region of L gene of *Watermelon silver mottle virus* (WSMoV), for resistance to the homologous virus and two serologically unrelated tospovirus species of *Tomato spotted wilt virus* (TSWV) and *Groundnut ringspot virus* (GRSV) under greenhouse conditions.

Transgene	No. of line assayed	Challenged with			TSWV-HR lines challenged with		
		TSWV			WSMoV	GRSV	
		HR^a^	MR^b^	S^c^	HR	HR	MR
WLm	94	5 (5.3%)	25 (26.6%)	64	5 (100%)	2 (40%)	2 (40%)
WLmt	82	7 (8.5%)	23 (28%)	52	7 (100%)	3 (42.9%)	3 (42.9%)
WLmts	88	7 (8%)	23 (26.1%)	58	7 (100%)	3 (42.9%)	3 (42.9%)
WLmA	76	9 (11.8%)	21 (27.6%)	36	9 (100%)	5 (55.6%)	3 (33.3%)
WLmhp	78	12 (15.4%)	18 (23.1%)	38	12 (100%)	7 (58.3%)	3 (25%)

a Transgenic tomato lines exhibited no symptoms; lines that were ELISA negative for TSWV NP were regarded as highly resistant (HR) lines.

b Transgenic tomato lines exhibited delayed systemic symptoms for more than 7 days as compared with non-transgenic control were classified as moderately resistant (MR) lines.

c Transgenic tomato lines that exhibited typical systemic symptoms similar to non-transformed plants after inoculation with TSWV at the same time or less than 7-day delay as compared to the non-transgenic control were classified as susceptible (S) lines.

**Figure 5:**
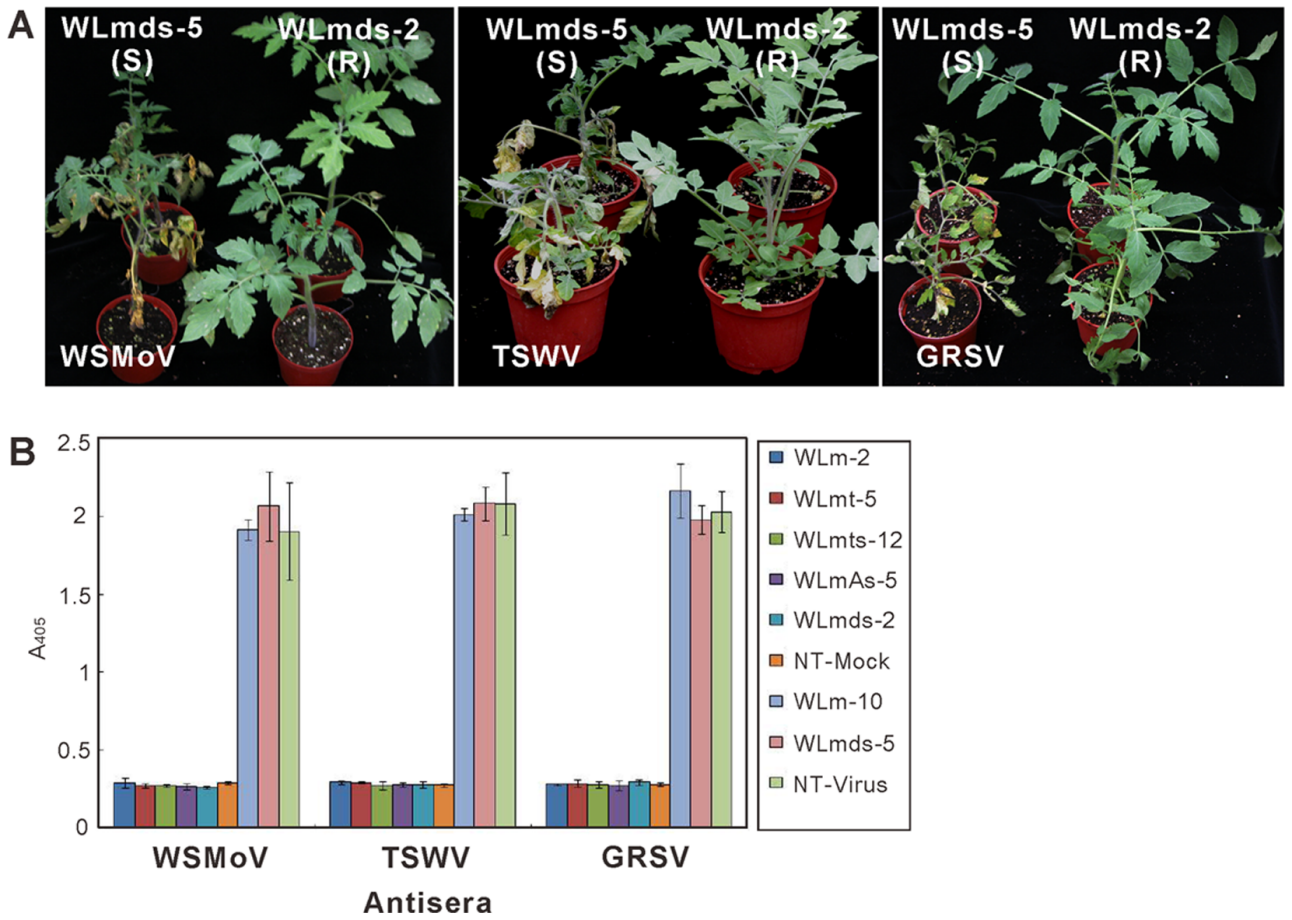
Evaluation of the transgenic tomato lines for resistance to distinct tospovirus species under greenhouse conditions. All transgenic lines carried the double-stranded inverted repeat of conserved region of L gene (WLmhp) of *Watermelon silver mottle virus* (WSMoV). The photograph was taken and the indirect enzyme-linked immunosorbent assay (ELISA) was conducted 14 days post-inoculation. (A) Resistant (R) line WLmhp-2 exhibited broad-spectrum resistance to three tospovirus species, including WSMoV, *Tomato spotted wilt virus* (TSWV) and *Groundnut ringspot virus* (GRSV). Susceptible (S) line WLmhp-5, which was included as a control, showed typical systemic symptoms similar to the non-transgenic control after inoculation with these three tospoviruses. (B) Virus infections were confirmed by indirect ELISA using the antiserum against the nucleocapsid protein of WSMoV, TSWV or GRSV. ELISA readings were recorded from the average of three independent experiments.

The virus infection of symptomatic transgenic tomato plants (Figure 5) was confirmed by indirect ELISA using the antiserum against the N protein of WSMoV, TSWV or GRSV. No significant accumulation of the virus was detected in the systemic leaves of symptomless plants, indicating that the resistant transgenic plants confer complete resistance to virus infection (Figure 5). Taken together, our results demonstrated that the L gene conserved region of WSMoV is able to trigger broad-spectrum resistance not only against the homologous WSMoV, but also against other serologically unrelated tospoviruses, such as TSWV and GRSV, in transgenic tomato plants.

### Broad-spectrum Resistant Transgenic Tobacco Lines Carrying One to Multiple Copies of Transgenes

**Figure 6:**
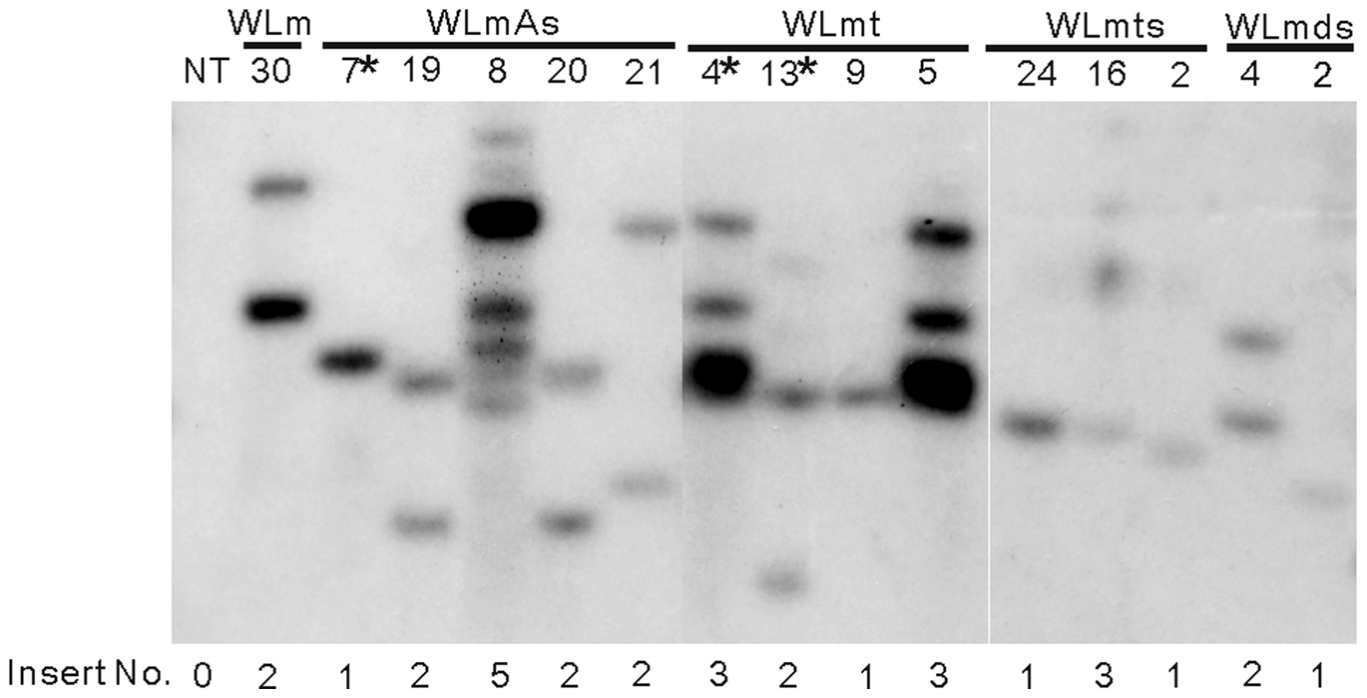
Detection of transgene insert numbers in the transgenic *Nicotiana benthamiana* R_0_ lines. (A) The insert numbers of individual transgenes are indicated. A non-transgenic (NT) *N. benthamiana* plant was used as a negative control.

The genomic DNAs isolated from the leaves of transgenic tobacco plants exhibiting broad-spectrum resistance against five different tospovirus species, including WLm-30, WLmA-7, 8, 19, 20 and 21, WLmt-4, 5, 9 and 13, WLmts-2, 16 and 24, and WLmhp-2 and 4, were digested with *Ssp*I. Results of hybridization revealed that these resistant transgenic tobacco lines carried one to seven copies of transgenes as shown in Figure 6. The similar patterns observed for WLmA-19 and WLmA-20 suggested that they might have originated from a same transformed cell. The particular lines WLmA-7, WLmt-4 and WLmt-13, which exhibited immunity to 10 different tospoviruses, contain 1–3 inserts of transgenes. Hence, our result indicated that the broad-spectrum resistance is not correlated to the insert number of transgenes.

### Broad-spectrum Resistance is Mediated by RNA Silencing

**Figure 7:**
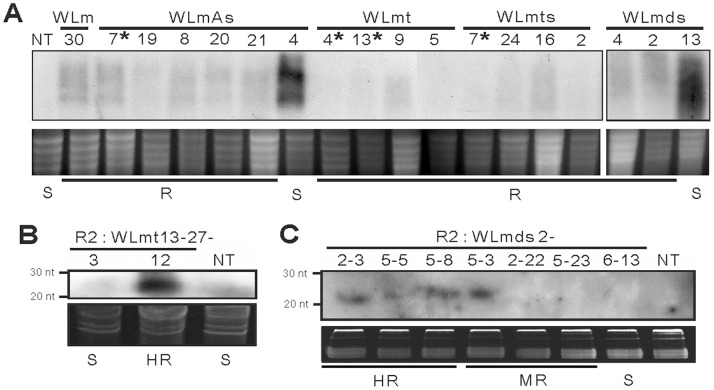
Detection of transgene transcripts in R_0_ lines of transgenic *Nicotiana benthamiana* and siRNA accumulation in R_2_ progeny of *N. bentamiana* and tomato lines resistant to *Watermelon silver mottle virus* (WSMoV). (**A**) All the analyzed resistant (R) lines showed lower levels of transgene transcripts than the susceptible (S) lines. The transgenic *N. benthamiana* lines completely resistant to 10 distinct tospovirus species are indicated by asterisks. Transgene transcripts were not detected in the NT plant of *N. benthamiana*. Ribosomal RNAs were used as loading controls. (**B**) siRNA accumulation of a susceptible (S) R_2_ individual (WLmt 13-27-3) and a highly resistant (HR) R_2_ individual (WLmt 13-27-12) of a tobacco line immune to WSMoV, in comparison to a non-transgenic (NT) plant, was analyzed by ^32^P-labeled WLm-specific probe. (**C**) siRNA accumulation of R_2_ progeny of a HR tomato line WLmhp 2. Accumulation of siRNA in R_2_ HR individuals (WLmhp 2-2-3, 2-5-5 and 2-5-8), moderately resistant (MR) individuals (WLmhp 2-5-3, 2-2-22 and 2-5-23), an S individual (WLmhp 2-6-13), and a NT plant were analyzed by ^32^P-labeled WLmhp-specific probe.

Significant amounts of transgene transcript were detected from samples from *N. benthamiana* plants of two randomly selected susceptible transgenic lines, WLmA-4 and WLmhp-13 (Figure 7A). Steady-state levels of transgene transcripts were not detected in all the assayed transgenic *N. benthamiana* samples conferring broad-spectrum resistance to five different tospovirus species, including WLm-30, WLmA-7, 8, 19, and 21, WLmt-4, 5, 9 and 13, WLmts-2, 7, 16 and 24, and WLmhp-2 and 4 (Figure 7A).

Since transgenic tobacco and tomato lines were obtained at different times, siRNA analyses were conducted on the R_2_ population of selected HR tobacco or tomato lines. High accumulation of siRNA was observed in the progeny of the highly resistant tobacco line WLmt13, the 21 nt siRNA was found in the resistant individual WLmt-13-27-12, but not present in the susceptible individual WLmt-13-27-3 (Figure 7B). Also, when the R_2_ progeny of the HR tomato line WLmhp-2 were analyzed, the 21 nt siRNA was found present in the HR individuals of WLmhp-2-2-3, 2-5-5, and 2-5-8, and WR individual of WLmhp-2-5-3, whereas no siRNA accumulation was detected in the two WR and one susceptible individuals (Figure 7C). Taken together, the results of transcript silencing and siRNA accumulation were correlated to the degrees of the transgenic resistance, indicating that the broad-spectrum resistance conferred by the L gene conserved region-transgenic tobacco plants is mediated by PTGS.

## Discussion

The effectiveness of PDR in terms of strength, spectrum and durability is largely depends upon the selection of appropriate segment of pathogen genome and its engineering into an effective transgene. In the present study, the tospovirus L gene region containing the highly conserved five RdRp motifs [16], [43], [46], [47] was amplified from WSMoV L gene (nt 3975 to 4928 of vc strand of WSMoV L gene) and designed into various transgenes that can trigger PTGS in the transformants of the model plant *N. benthamiana* and the real crop tomato. When the resistance levels of transgenic *N. benthamiana* plants were compared, the transgenic lines carrying individual transgenes were able to provide complete resistance to the homologous virus WSMoV and also to serologically unrelated 9 heterologous tospovirus species of different phylogenetic clades of tospoviruses. However, the resistance is not extended to unrelated viruses like TuMV and CMV that do not belong to the genus *Tospovirus*, indicating that the broad-spectrum resistance is *Tospovirus* genus-specific. The broad-spectrum resistance was further evidenced in transgenic tomato lines which provide complete resistance to the homologous and serologically unrelated heterologous tospovirus species.

The transgene transcripts were not detected in all the tested resistant transgenic *N. benthamiana* lines, while they were detectable in the susceptible lines. Furthermore, siRNA accumulation was detected in the resistant R_2_ individuals of the selected tobacco and tomato HR lines, but not in the susceptible R_2_ individuals. These results demonstrated that PTGS is the underlying the mechanism for resistance. The non-translatable transgenes of WLmt, WLmts, WLmA and WLmhp effectively triggered PTGS, as described previously for RNA-mediated transgenic resistance [67], [68].

Some studies reported that translatability of transgenes was necessary for replicase-mediated resistance to TMV, *Pea early browning virus* and CMV [69], [70], [71]. The CMV-resistant tobacco lines containing translatable 2a replicase transgenes exhibited either delay in symptom development or complete resistance [71]. It was considered that replication of CMV was interfered and the cell-to-cell and/or long-distance movement of the virus was restricted through unidentified cellular processes [72], [73], [74], [75]. However, the effectiveness of transgene-induced silencing could not be ruled out in these previous studies, because some resistant lines containing translatable transgenes also generated RNA-mediated resistance correlating with low levels of accumulation of transgene mRNA [71]. In the present study, similar replicase-mediated effect from the translatable construct WLm could not be excluded due to the lack of specific antibody for verifying translation from WLm. However, lower transgene transcript levels observed with the translatable WLm construct (Figure 7A) suggest that PTGS is the main underlying resistance mechanism.

PTGS is sequence homology-dependent. The WLm region within L genes of TSWV and INSV show 69% and 66.4% nucleotide identity, respectively, to that of WSMoV. This level of identity (66–69%) is lower than the level of identity (∼88%) considered being required for effective targeting for PTGS [76]. However, the contexts of L genes of TSWV and INSV corresponding to the highly conserved five RdRp motifs of L protein share higher identities of 78–85% and 76–86%, respectively, with those of WSMoV L gene. This level of identity in combination with the relatively less mutable nature of RdRp motifs may explain the observed PTGS-mediated resistance of WLm transgenic plants to the serologically unrelated tospoviruses. The DNA probe corresponding to the conserved region of WSMoV L gene was able to hybridize with total RNAs extracted from plant tissues infected with TSWV and INSV, suggesting that the WLm transgenes are also able to interact with the L genes of tospoviruses not belonging to the WSMoV clade (Figure 2B). Corroboratively, hybridization signals were not detected for TuMV (*Potyvirus*) and CMV (*Cucumovirus*). Challenge assay with TuMV and CMV validated the results of hybridization. Taken together, the results of hybridization analysis and challenge assay suggest that the broad-spectrum transgenic resistance mediated by WSMoV L gene RdRp motifs is specific against the genus *Tospovirus*.

Although some of the full-length L RNAs of the other challenged tospoviruses have not been determined yet, the transgenic resistance against these viruses logically demonstrates that the L RNAs of GRSV and GCFSV share homology with those of WSMoV, TSWV and INSV, as described in our previous reports [43], [46]. The conserved region of the L RNAs of MYSV, GBNV and CaCV share high identities of 79.6% to 89.9%, 81.3% to 88.2% and 77.8% to 93.8%, respectively, with the five RdRp motifs of WSMoV (Table 1). Thus, it is not surprising that the L gene conserved region-transgenic tobacco plants also provide resistance against these three economically important Asia-type tospoviruses, and other Asia-type tospovirus species of WBNV, TYRV and IYSV, which belong to WSMoV and IYSV clades [16], [47], [77], [78]. The tospovirus N protein dendrogram (Figure 4) highlighting different clades and tested viruses illustrates the *Tospovirus* genus level broad spectrum resistance, though we are yet to test the members of Bean necrosis mosaic virus (BeNMV)-Soybean vein necrosis-associated virus (SVNaV) clade, which was discerned recently by phylogenetic analyses with tospoviral protein sequences [79]. SVNaV, one of the known two members of the BeNMV-SVNaV clade [79], was shown to be a distinct serotype by analyzing its serological relationship with GRSV, INSV, TCSV, TSWV and TYRV [80].

The negative sense viral (v) L RNA of tospoviruses is the template for L RNA replication and transcription. The transcribed positive sense viral complementary (vc) L RNA is the messenger RNA for expressing replicase protein [4]. Both v and vc L RNAs, believed to be present simultaneously in the infected cells, are possibly targeted by the siRNAs processed from the transcripts, resulting in inhibition of L RNA replication and translation.

The members of the genus *Tospovirus* are distributed worldwide and they cause severe damage to many economically important crops. Wide genetic variations of tospoviruses and their persistent transmission by thrips make the diseases caused by tospoviruses difficult to be controlled. Sources of natural resistant genes to *Tospovirus* spp. suitable for commercial breeding are very limited and extensive studies have been made to create engineered resistance against these viruses. Several studies have shown that transgenic plants can confer broad-spectrum resistance against distinct tospovirus species. For instance, transgenic *N. benthamiana* plants expressing the N protein-interacting peptide derived from the N ORF of TSWV generated high levels of resistance to TSWV, GRSV, TCSV and CSNV [35] belonging to TSWV clade.

A strategy using a single transgene by combining small RNA fragments from N genes of WSMoV, TSWV, GRSV and TCSV in hairpin construct to trigger RNA silencing for generating a high frequency broad-spectrum resistance against all the four tospoviruses was reported [34]. However, this approach is N gene homology-dependent and difficult to be applied for controlling the different tospoviral strains or newly emergent species in the genus *Tospovirus*. Hassani-Mehraban *et al*. [81] generated transgenic *N. benthamiana* plants resistant to five different tospoviruses, i.e., WSMoV, TSWV, GRSV, TCSV and TYRV-t (tomato-infecting strain of TYRV) using an inverted repeat construct containing partial N gene sequences from these viruses. However, the transgenic resistance against TYRV-t does not hold against the soybean-infecting strain TYRV-s; the resistance against TYRV-s was broken down by TYRV-t, when it was co-inoculated with TYRV-s [81].

We have first reported that an alternative approach that the 21 nt viral sequences of the conserved motif of a viral PTGS suppressor gene constructed as amiRNAs is able to confer complete resistance to virus infection in transgenic *Arabidopsis* plants [65]. Our further effort revealed that amiRNAs precisely targeting at the conserved RdRp motifs of WSMoV L gene, individually or in different combination, confer high degrees of transgenic resistance against the homologous virus, but not against different tospovirus species with 2 or 3 mismatches in the targeted RdRp motifs in the same WSMoV clade [32].

Differing from all the aforementioned approaches for generating transgenic resistance to tospoviruses, here we have successfully developed a novel transgenic approach using a single fragment from the L RNA of WSMoV, which encompasses all five highly conserved RdRp motifs in their native contiguous context, as transgenes to generate transgenic tobacco and tomato plants conferring broad-spectrum resistance against distinct tospovirus species at the genus level. In earlier reported tospovirus N gene transgenic plants, the N gene-mediated resistance was restricted to homologous or closely related viruses [25], [27], [30], [31], owing to relatively less conserved nature of N gene sequences. The *Tospovirus* genus level resistance of the present WLm transgenic *N. benthamiana* and tomato plants can be attributed to higher degree of conservation of L gene and relatively non-mutable nature of L gene sequence contexts corresponding to the essential RdRp motifs of L protein. The superior performance of WLmhp transgene to other transgenes may partly be due to the hairpin structure of the transcribed RNA and the presence of the eight conserved terminal nucleotides of tospoviral RNAs as the linker connecting the two arms of the hairpin. However, the possible contribution of the eight terminal nucleotides of tospoviral RNAs to the genus level resistance conferred by WLmhp transgene was not assessable because of the lack of an appropriate control. Our transgenic approach should provide a more practical and durable measure for controlling the devastating diseases caused by the prevailing and newly emerging tospoviral strains or species in different regions of the world.
